# Macrophage migration inhibitory factor facilitates prostaglandin E_2_ production of astrocytes to tune inflammatory milieu following spinal cord injury

**DOI:** 10.1186/s12974-019-1468-6

**Published:** 2019-04-13

**Authors:** Yuxin Zhang, Yue Zhou, Shuxia Chen, Yuming Hu, Zhenjie Zhu, Yingjie Wang, Nan Du, Tiancheng Song, Yumin Yang, Aisong Guo, Yongjun Wang

**Affiliations:** 1 Key Laboratory of Neuroregeneration of Jiangsu and Ministry of Education, Co-innovation Center of Neuroregeneration, Nantong University, 19 Qixiu Road, Nantong, 226001 People’s Republic of China; 2 Department of Rehabilitation Medicine, Affiliated Hospital of Nantong University, Nantong, 226001 People’s Republic of China; 3 Department of Pediatrics, Yancheng City No.1 People’s Hospital, Yancheng, 224005 People’s Republic of China

**Keywords:** MIF, COX2, Spinal cord, Astrocyte, Inflammation, PGE_2_

## Abstract

**Background:**

Astrocytes have been shown to produce several pro- and anti-inflammatory cytokines to maintain homeostasis of microenvironment in response to vast array of CNS insults. Some inflammation-related cytokines are responsible for regulating such cell events. Macrophage migration inhibitory factor (MIF) is a proinflammatory cytokine that can be inducibly expressed in the lesioned spinal cord. Unknown is whether MIF can facilitate the production of immunosuppressive factors from astrocytes to tune milieu following spinal cord injury.

**Methods:**

Following establishment of contusion SCI rat model, correlation of PGE_2_ synthesis-related protein levels with that of MIF was assayed by Western blot. ELISA assay was used to detect production of PGE_2_, TNF-α, IL-1β, and IL-6. Immunohistochemistry was performed to observe colocalization of COX2 with GFAP- and S100β-positive astrocytes. The primary astrocytes were treated by various inhibitors to validate relevant signal pathway.

**Results:**

The protein levels of MIF and COX2, but not of COX1, synchronously increased following spinal cord injury. Treatment of MIF inhibitor 4-IPP to the lesion sites significantly reduced the expression of COX2, mPGES-1, and as a consequence, the production of PGE_2_. Astrocytes responded robustly to the MIF interference, by which regulated MAPK/COX2/PGE_2_ signal pathway through coupling with the CD74 membrane receptor. MIF-induced production of PGE_2_ from astrocytes was able to suppress production of TNF-α, but boosted production of IL-1β and IL-6 in LPS-activated macrophages.

**Conclusion:**

Collectively, these results reveal a novel function of MIF-mediated astrocytes, which fine-tune inflammatory microenvironment to maintain homeostasis. These suggest an alternative therapeutic strategy for CNS inflammation.

**Electronic supplementary material:**

The online version of this article (10.1186/s12974-019-1468-6) contains supplementary material, which is available to authorized users.

## Background

Spinal cord injury (SCI) always initiates a robust inflammatory response characterized by infiltration of leukocytes and synthesis of cytokines and chemokines [1, 2]. This excessive inflammation induced by SCI causes a progressive damage through aggravating the degeneration of neurons and apoptosis of oligodendrocytes [3]. The formation of inflammatory cascades in the central nervous system (CNS) is considered as a result of tightly coordinated interaction between parenchymal cells (neurons and glia), endothelia, and circulating leukocytes [2, 4]. Damage-associated molecular patterns (DAMPs) released from injured and dying cells, or from the circulation, activated the pattern-recognition receptors (PRRs) of leukocytes, microglia, and astrocytes to drive the inflammatory response in the absence of infection, thereby affect tissue functional recovery [5, 6]. Astrocytes are one of the first cell populations to sense SCI, and subsequently participate in modulation of inflammatory responses [7, 8]. On the one hand, the astrocytes express several toll-like receptors (TLR) and build up responses to innate immune triggers by releasing proinflammatory molecules that exacerbate spinal cord injuries [9, 10]. On the other hand, they can also limit the spread of inflammatory cytokines or injury-induced toxic molecules through physical glial scar and/or secretion of anti-inflammatory cytokines. For example, anti-inflammatory cytokine IL-10 can be released by human astrocyte cell lines stimulated with IFN-γ1a or combinations of proinflammatory cytokines (LPS/IFN-γ) plus IFN-γ1a [11]. Stimulation of TLR3 induces anti-inflammatory cytokines in adult human astrocytes, and observation was recapitulated by applying LPS to neonatal rats, which decreased proinflammatory responses in EAE at later age, as measured by increased IL-10 in the spinal cord [12–14]. The differential outcomes of astrocytes have been shown to tightly depend on injury modes of CNS and diversity of stimulating factors [14–16]. Obviously, astrocytes are required for the fine-tuned regulation and resolution of the inflammation and subsequent reconstruction of microenvironment homeostasis following SCI [15].

Cyclooxygenases (COX), also known as prostaglandin-endoperoxide synthases (PTGS), catalyze the reaction of arachidonic acid (AA) metabolism to prostanoids and thromboxanes [17]. COX1 and COX2 are the two main COX enzymes that participate in such catalytic process. Several bioactive lipid mediators produced in the AA metabolism have been found to play crucial roles in inflammation, thus COX1 and COX2 are widely used as targets of the nonsteroidal anti-inflammatory drugs [18]. COX1 is constitutively expressed in most tissues to maintain essential physiological functions including platelet homeostasis, gastric mucosal integrity, and neuronal function [19, 20], while COX2 is an inducible enzyme that responds to proinflammatory stimuli [17]. Since COX2 is expressed in the normal CNS, but is rare or absent in most organs, injury-induced COX2 expression has been shown to exacerbate neuronal death and neuroinflammatory responses of CNS [21, 22]. The mechanism by which COX2 modulates inflammation is poor defined, but possibly be associated with an increase in the production of prostanoids like prostaglandin E2 (PGE_2_) [18]. During the initial stage of inflammatory responses, PGE_2_ acts as a proinflammatory mediator by promotion of local vasodilatation, inflammatory edema, and fever [23]. However, it also exhibits immunosuppressive properties through contributing to the resolution phase of acute inflammation [24]. For instance, PGE_2_ has been shown to inhibit production of TNF-α and boost the production of IL-10 in macrophages [25]. Thus, PGE_2_ potentially facilitates the return of tissue microenvironment to homeostasis in the case of tissue pathology. In the CNS, COX2 is localized primarily within neurons, but it can be inducibly expressed in the astrocytes and microglia following CNS injury [17, 26, 27]. Whether COX2-induced production of PGE_2_ from astrocytes contributes to the microenvironment homeostasis following SCI is unclear.

Macrophage migration inhibitory factor (MIF) is a proinflammatory cytokine produced by a variety of cells and tissues, including monocytes, macrophages, T and B lymphocytes, and hepatocytes [28]. In the CNS, MIF is able to be inducibly expressed within neurons, astrocytes, and microglia, in association with neuronal apoptosis and facilitation of neuroinflammation [29–31]. Therefore, MIF is supposed to be a potential therapeutic target for several autoimmune-mediated CNS diseases such as multiple sclerosis (MS) [31]. It is interesting to note that MIF is capable of promoting the release of chemokine CCL5 from astrocytes, which preferentially attracts M2-type macrophages toward the lesion sites of spinal cord [30]. The accumulation of M2-type macrophages has been shown to be tightly coordinated with the resolution of inflammation, hinting at a function of MIF in tune the microenvironment through astrocytes. Since MIF has been shown to activate COX2/PGE_2_ signaling in spinal microglia, and the synthesis of PGE_2_ in astrocytes is also induced by proinflammatory stimuli [27, 32], it is assumed that MIF can exert the same function to induce the PGE_2_ production from astrocytes, which in turn regulates the activities of inflammatory cells. In the present study, we examined the relevance between expression of MIF and PGE_2_ following rat spinal cord contusion. We further investigated the mechanism of MIF-induced production of PGE_2_ in the astrocytes, as well as its effects on the inflammatory responses of macrophages. Our results have revealed that MIF can tune inflammatory microenvironment of injured spinal cord through influence of astrocytes.

## Methods

### Animals

Adult male Sprague–Dawley (SD) rats, weighing 180–220 g, were provided by the Center of Experimental Animals, Nantong University. All animal care, breeding, and testing procedures were approved according to the Animal Care and Use Committee of Nantong University and the Jiangsu Province Animal Care Ethics Committee. All animals were housed in individual cages in a temperature and light/dark cycle controlled environment with free access to food and water.

### Establishment of contusion SCI rat model

The number of animals subjected to surgical treatment was calculated by six per experimental group in triplicate. Contusion SCI rat model was prepared as previous description [33]. Briefly, rats were anesthetized with an intraperitoneal injection of 10% chloral hydrate compound anesthetic (3 mg/kg). The fur was shaved from the surgical site and the skin was disinfected with chlorhexidine. A 15-mm midline skin incision was made to expose the vertebral column. After the spinal thoracic region was exposed by separation of dorsal muscles to the side, the spinous processes of T8–T10 vertebrae were exposed. A laminectomy was performed at vertebral level T9, exposing the dorsal cord surface with the dura remaining intact. The exposed spinal cord segment (about 3 mm in length) received a 150-kilodyne spinal contusion injury using the IH-0400 Impactor (Precision Systems and Instrumentation) injury device. The impact rod was removed immediately, and the wound was irrigated. For drug delivery, 10 μl of 80 mM COX2 inhibitor NS398 or vehicle were then slowly injected intrathecally. Muscles and incisions were sutured using silk threads. Postoperative care included butorphanol administration twice a day for a 5-day period, as well as vitamins, saline, and enrofloxacin twice a day for a 7-day period. Manual expression of bladders was performed twice a day until animals recovered spontaneous voiding.

### Cell culture

Astrocytes were prepared from spinal cord of newborn Sprague–Dawley rats, 1–2 days after birth for maximum homogeneity, and the astrocytes were isolated and cultured according to previously described methods [34]. Briefly, the cells were enzymatically dissociated using 0.25% trypsin (Gibco-BRL) for 6 min at 37 °C, and the suspension was then centrifuged at 1200 rpm for 5 min and cultured in 1:1 Dulbecco’s modified Eagle’s medium:Ham’s F-12 medium supplemented with 10% fetal bovine serum (FBS), 0.224% NaHCO_3_, and 1% penicillin/streptomycin in the presence of 5% CO_2_. A monolayer of astrocytes was obtained 12–14 days after the plating. Non-astrocytes were detached from the flasks by shaking and were removed by changing the medium. Third or fourth passage cells were rendered quiescent through incubation in medium containing 0.5% FBS for 4 days prior to the experiments. Astrocyte phenotype was confirmed by cells exhibiting a characteristic morphology and positive staining for the astrocytic marker glial fibrillary acid protein (GFAP).

For the culture of macrophages in the astrocyte-conditioned medium, the isolated astrocytes were cultured in the presence of 1 μg/ml recombinant MIF with or without addition of 30 μM NS398 for 24 h. Meanwhile, astrocyte-free culture was used as negative control. The astrocyte-conditioned medium was then collected for the culture of macrophages in the presence of 1 μg/ml LPS at 24 h, respectively.

### Western blot

Protein was extracted from cells with a buffer containing 1% SDS, 100 mM Tris-HCl, 1 mM PMSF, and 0.1 mM β-mercaptoethanol, following treatment with 0–2.5 μg/ml rat recombinant MIF (ProSpec) for 24 h. Alternatively, protein was extracted from 1 cm spinal segments of injured site at 0 day, 1 day, 4 days, and 1 week following contusion (*n* = 8 in each time point). Protein concentration of each specimen was detected by the Bradford method to maintain the same loads. Protein extracts were heat denatured at 95 °C for 5 min, electrophoretically separated on 10% SDS-PAGE, and transferred to PVDF membranes. The membranes were subjected to the reaction with a 1:1000 dilution of primary antibodies in TBS buffer at 4 °C overnight, followed by a reaction with secondary antibody conjugated with goat anti-rabbit or goat anti-mouse HRP dilution 1:1000 (Santa Cruz) at room temperature for 2 h. After the membrane was washed, the HRP activity was detected using an ECL kit. The image was scanned with a GS800 Densitometer Scanner (Bio-Rad), and the data were analyzed using PDQuest 7.2.0 software (Bio-Rad). β-actin (1:5000) was used as an internal control. Antibodies used in Western blot are MIF, COX1, cPGES (Abcam); COX2, mPGES-1, mPGES-2 (Cayman); CD74 (Biorbyt); and β-actin (Proteintech).

### ELISA

Primary astrocytes were treated with 0–2.5 μg/ml rat recombinant MIF for 24 h. Cell supernatants were harvested, and cells were lysed in the buffer containing 1% SDS, 100 mM Tris-HCl, 1 mM PMSF, and 0.1 mM β-mercaptoethanol. The lysates were centrifuged at 12,000×*g* for 15 min. Levels of PGE_2_ (ARBOR ASSAYS), TNF-α, IL-1β, IL-6, IL-10, and IL-4 (MULTI SCIENCES) were assessed using the appropriate ELISA kits according to the manufacturer’s directions. Plates were read using a 96-well plate reader (Biotek Synergy2) at a 450 nm wavelength.

For determination of PGE_2_ and cytokines levels in the tissue samples, protein from 1 cm spinal segments of injured site at 0 day, 1 day, 4 days, and 1 week following contusion (*n* = 6 in each time point) was extracted with a buffer containing 1% SDS, 100 mM Tris-HCl, 1 mM PMSF, and 0.1 mM β-mercaptoethanol, respectively. The total protein of each sample was then diluted to final concentration at 0.5 μg/μl by the buffer before detection by ELISA kits.

### Tissue immunohistochemistry

The vertebra segments were harvested from six experimental models of each time point, post-fixed, and sectioned. Sections were allowed to incubate with polyclonal COX2 antibody (1:100 dilution, Cayman), goat anti-IBA-1 antibody (1:200 dilution, Abcam), monoclonal mouse anti-S-100 (β-Subunit) antibody (1:1000 dilution, Sigma), or monoclonal mouse anti-human GFAP antibody (1:400 dilution, Sigma) at 4 °C for 36 h. The sections were further reacted with the FITC-labeled secondary antibody goat anti-mouse IgG (1:400 dilution, Gibco), the TRITC-labeled secondary antibody donkey anti-rabbit IgG (1:400 dilution, Gibco), or the 488-labeled secondary antibody donkey anti-goat IgG (1:400 dilution, Abcam) at 4 °C overnight, followed by observation under a confocal laser scanning microscope (Leica, Heidelberg, Germany).

### Quantitative real-time polymerase chain reaction

Total RNA was prepared with Trizol (Gibco, USA) from macrophage RAW 264.7 treated with 1 μg/ml LPS for 24 h. The first-strand cDNA was synthesized using Omniscript Reverse Transcription Kit (QIAGEN) in a 20 μl reaction system containing 2 μg total RNA, 0.2 U/μl M-MLV reverse transcriptase, 0.5 mM dNTP mix, and 1 μM Oligo-dT primer. The cDNA was diluted 1:5 before use in quantitative real-time polymerase chain reaction (Q-PCR) assays. The sequence-specific primers were designed and synthesized by Invitrogen (Shanghai, China). Primer pairs for EP1: forward primer 5′-AAG CAG GCT GGC GAC GAA C-3′, reverse primer 5′-CCA ACA GGC GAT AAT GGC ACA-3′; for EP2, forward primer 5′-CTC GGA GGT CCC ACT TTT-3′, reverse primer 5′-GCG GAT TGT CTG GCA GTA-3′; for EP3, forward primer 5′-CGG TTG AGC AAT GCA AGA CA-3′, reverse primer 5′-GGT GGA GCT GGA AGC ATA GT-3′; for EP4, forward primer 5′-GAC AGC CAG CCC ACA TAC-3′, reverse primer 5′-GCG TCC TTC TCC TCC ACT-3′. Q-PCR reactions were performed in a final volume of 20 μl (1 μl cDNA template and 19 μl Q-PCR reaction buffer containing 2.5 mmol/L MgCl_2_, 0.2 mmol/L dNTPs, anti-sense and sense primers 0.5 μmol/L, Taqman probe 0.4 μmol/L, DNA polymerase 0.2 μl, and 1 × DNA polymerase buffer). The Rotor-Gene 5 software (Corbett Research, Rotor-Gene, Australia) was used for real-time PCR analysis. Reactions were processed using one initial denaturation cycle at 94 °C for 5 min followed by 40 cycles of 94 °C for 30 s, 60 °C for 30 s, and 72 °C for 30 s. Fluorescence was recorded during each annealing step. At the end of each PCR run, data were automatically analyzed by the system and amplification plots obtained. MIF full-length plasmid was used to prepare standard curves and used as a specificity control for real-time PCR. The expression levels were normalized to an endogenous β-actin. In addition, a negative control without the first-strand cDNA was also performed.

### Statistical analysis

Statistical significance of differences between groups was analyzed by one-way analysis of variance (ANOVA) followed by Bonferroni’s post-hoc comparisons tests with SPSS 15.0 (SPSS, Chicago, IL, USA). Normality and homoscedasticity of the data were verified before any statistical analysis using Levene’s test. Statistical significance was set at *p* < 0.05.

## Results

### Activation of COX2/PGE_2_ signal pathway in the astrocytes is relevant to expression of MIF following SCI of rat

**Fig. 1:**
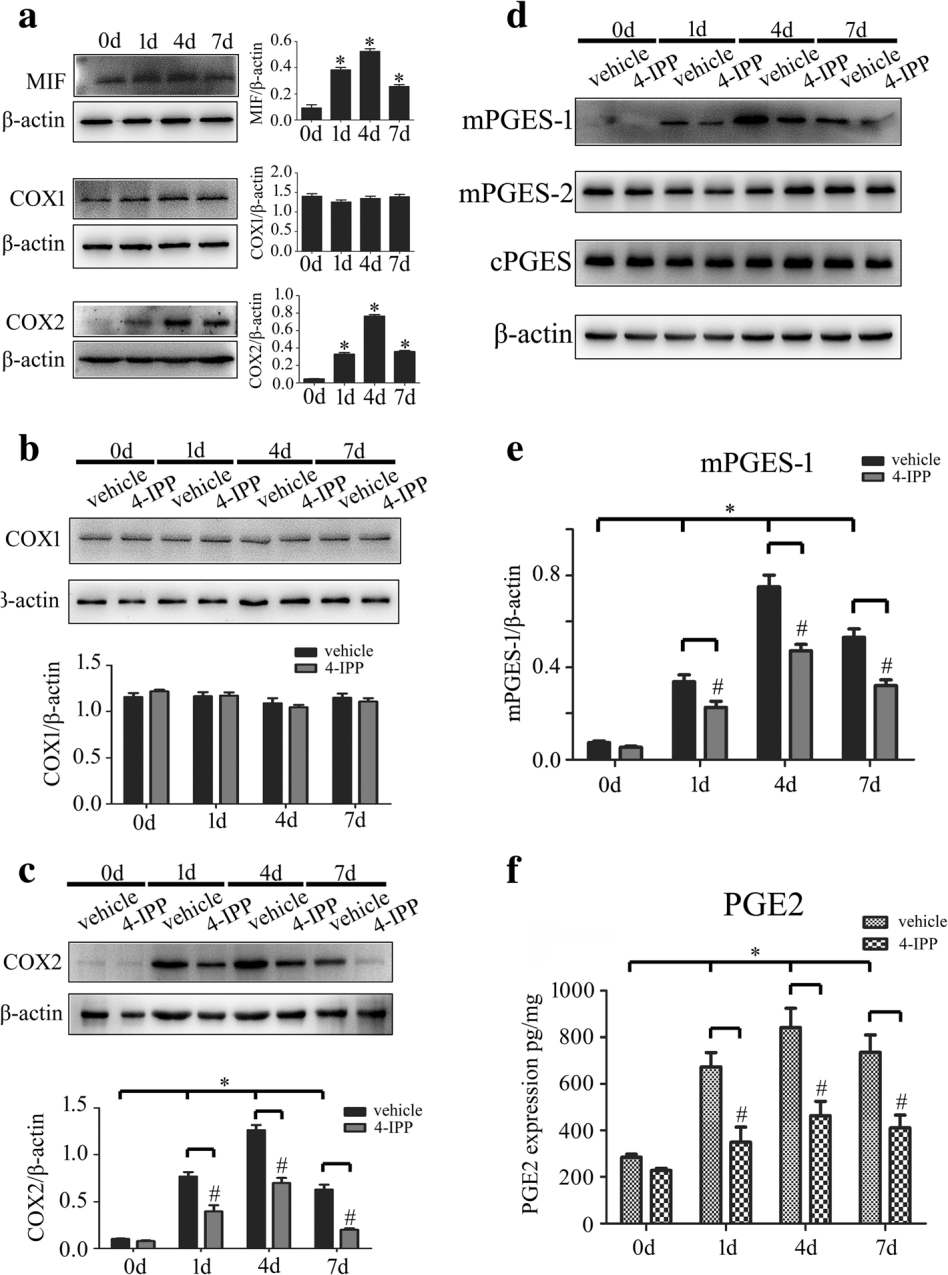
Determination of MIF protein levels and PGE_2_ production following spinal cord injury. **a** Western blot analysis of MIF, COX1, and COX2 protein levels following spinal cord contusion at 0 day, 1 day, 4 days, and 7 days, respectively. **b**–**e** Determination of COX1 (**b**), COX2 (**c**), mPGES-1, mPGES-2, and cPGES (**d**, **e**) protein levels for the injured cord at different time points with or without injection of 8 μl 4-IPP (100 mM) at the lesion sites. Quantities were normalized to endogenous β-actin. **f** Production of PGE_2_ was assayed by ELISA accordingly. Experiments were performed in triplicates. Error bars represent the standard deviation (*P < 0.05; #P < 0.05)

To examine whether MIF expression correlates to the PGE_2_ production of astrocytes following SCI, we firstly assayed protein levels of MIF, COX1, and COX2, as well as the isoforms of PGE_2_ synthase in the lesion sites following SCI at 0 day, 1 day, 4 days, and 7 days, respectively. Results showed that MIF, COX2, and microsomal PGE synthase-1 (mPGES-1), but not of COX1, mPGES-2, and cytosolic PGE synthase (cPGES), were inducibly expressed following SCI with a peak level at 4 days (Fig. 1a–e). Accordingly, the production of PGE_2_ increased synchronously, as detected by ELISA (Fig. 1f). Treatment of 8 μl of 100 mM MIF inhibitor 4-IPP at the lesion sites resulted in remarkable a decrease of COX2 and mPGES-1 protein levels (Fig. 1c–e), as well as a reduction of PGE_2_ production (Fig. 1f), whereas COX1, mPGES-2, and cPGES were kept unaffected (Fig. 1b, d).

**Fig. 2:**
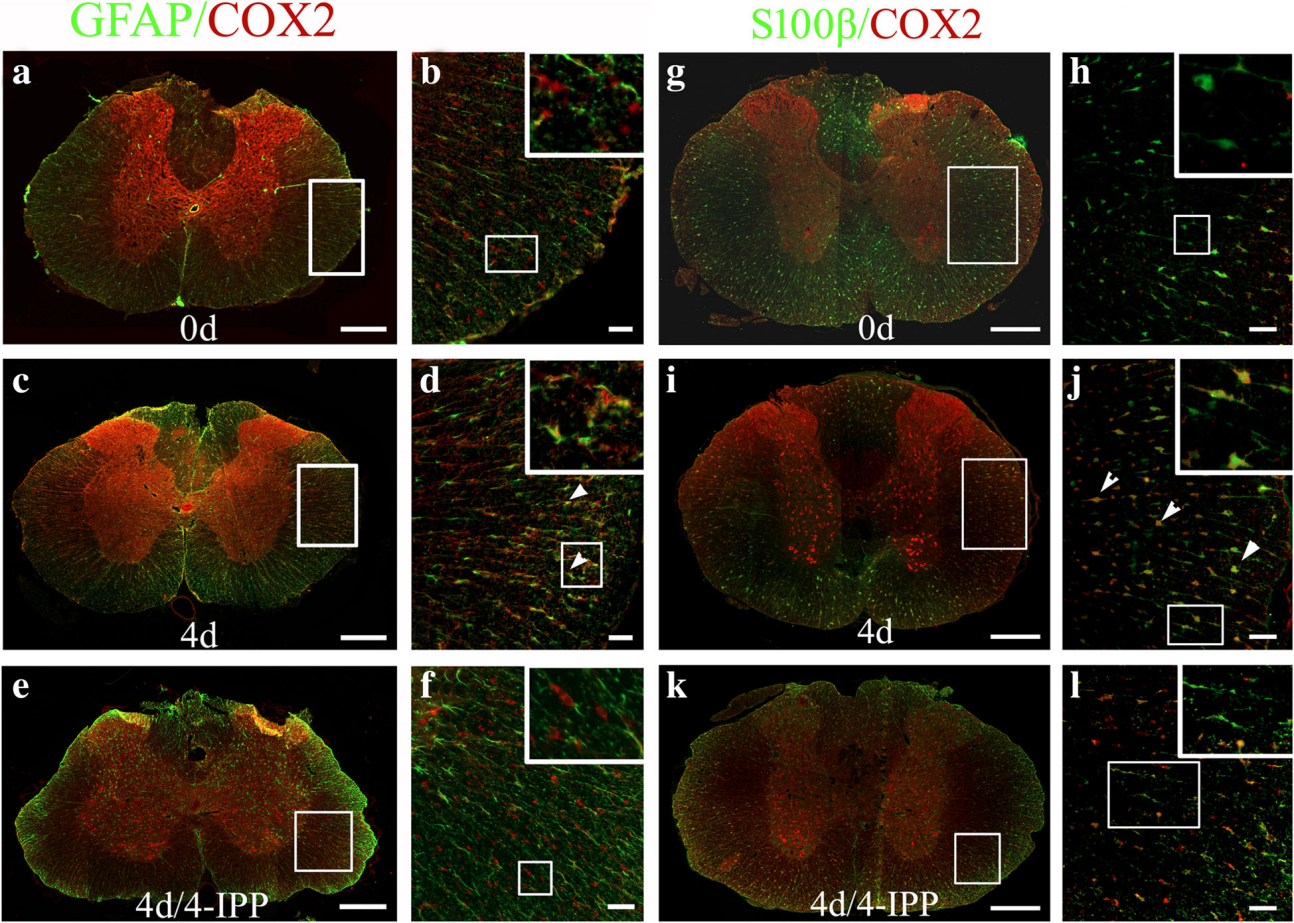
Colocalization of COX2 with astrocytes. **a**–**l** Immunostaining of COX2 in the cross sections of rat contused spinal cord showed colocalization with GFAP- (**a**–**f**) and S100β- (**g**–**l**) positive cells at 0 day and 4 days, with or without injection of 4-IPP, respectively. Rectangle indicates region magnified. Arrowheads indicate colocalization of COX2 with astrocytes. Scale bars, 500 μm in (**a**), (**c**), (**e**), (**g**), (**i**), and (**k**); 50 μm in (**b**), (**d**), (**f**), (**h**), (**j**), and (l)

Immunostaining was then performed to understand specific cell types that respond to MIF stimulation. As the roles of COX2 in neurons have been well described previously [22, 35], we therefore sought to detect its colocalization with astrocytes and microglia. Results demonstrated that COX2 expression was significantly induced in GFAP- and S100β-positive astrocytes at 4 days following SCI (Fig. 2a–d, g–j). Distinctively, expression of COX2 was moderately upregulated in IBA-1-positive microglia following SCI (Additional file 1: Figure S1). Treatment of 4-IPP attenuated the expression of COX2 in the astrocytes (Fig. 2e, f, k, l). These data indicate that injury-induced MIF is able to activate COX2/PGE_2_ signal pathway of astrocytes following SCI.

### MIF facilitated expression of COX2 and production of PGE_2_ in primary cultured astrocytes

**Fig. 3:**
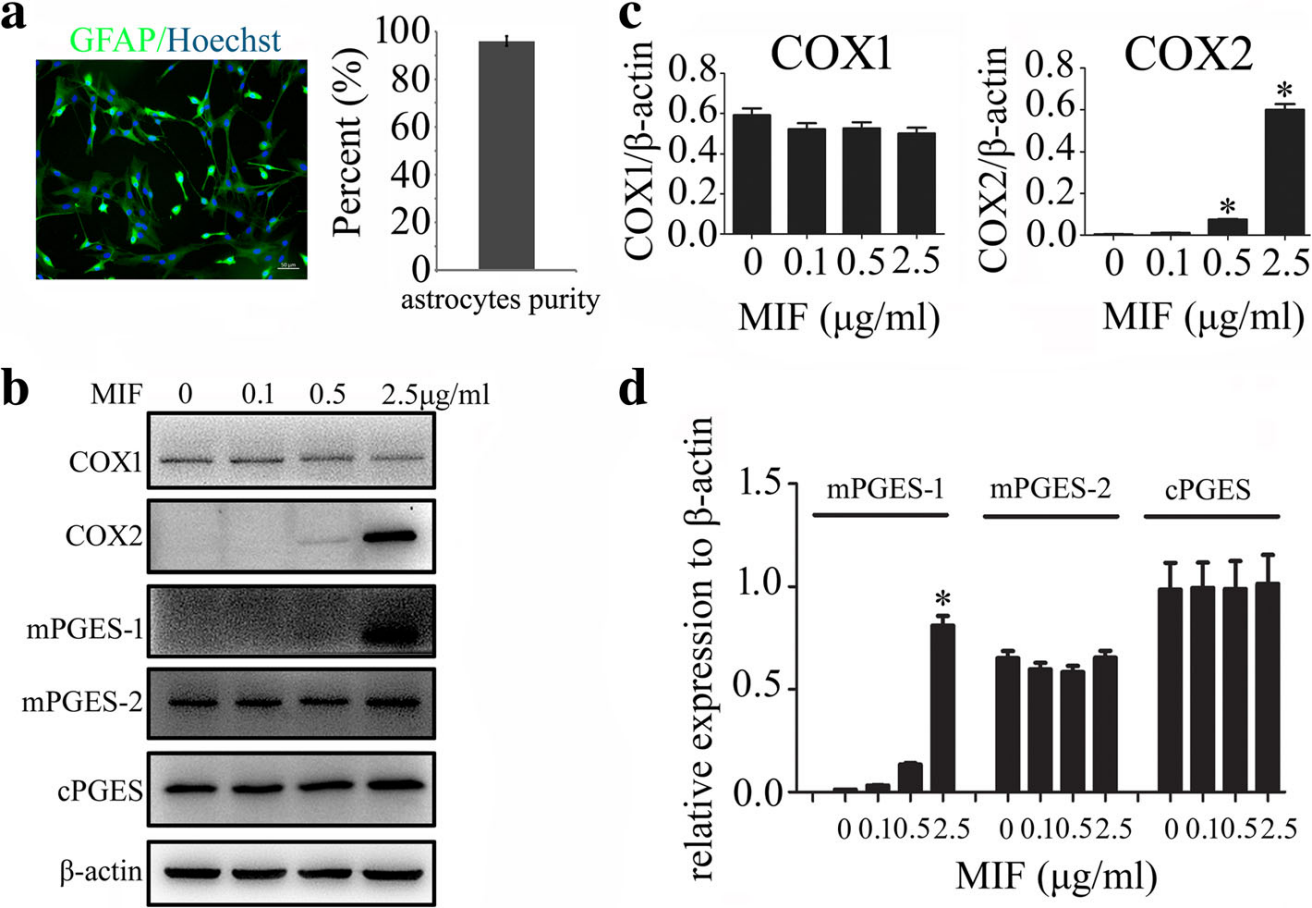
Determination of PGE_2_ synthesis-related protein levels in response to recombinant MIF stimulation. **a** Showing isolated astrocytes stained with GFAP and Hoechst 33342 with purity more than 95%. **b** Western blot analysis of COX1, COX2, mPGES-1, mPGES-2, and cPGES following astrocytes treatment with 0–2.5 μg/ml recombinant MIF for 24 h. **c**, **d** Quantification data as shown in (**b**). Experiments were performed in triplicates. Error bars represent the standard deviation (*P < 0.05). Scale bars, 50 μm in (**a**)

**Fig. 4:**
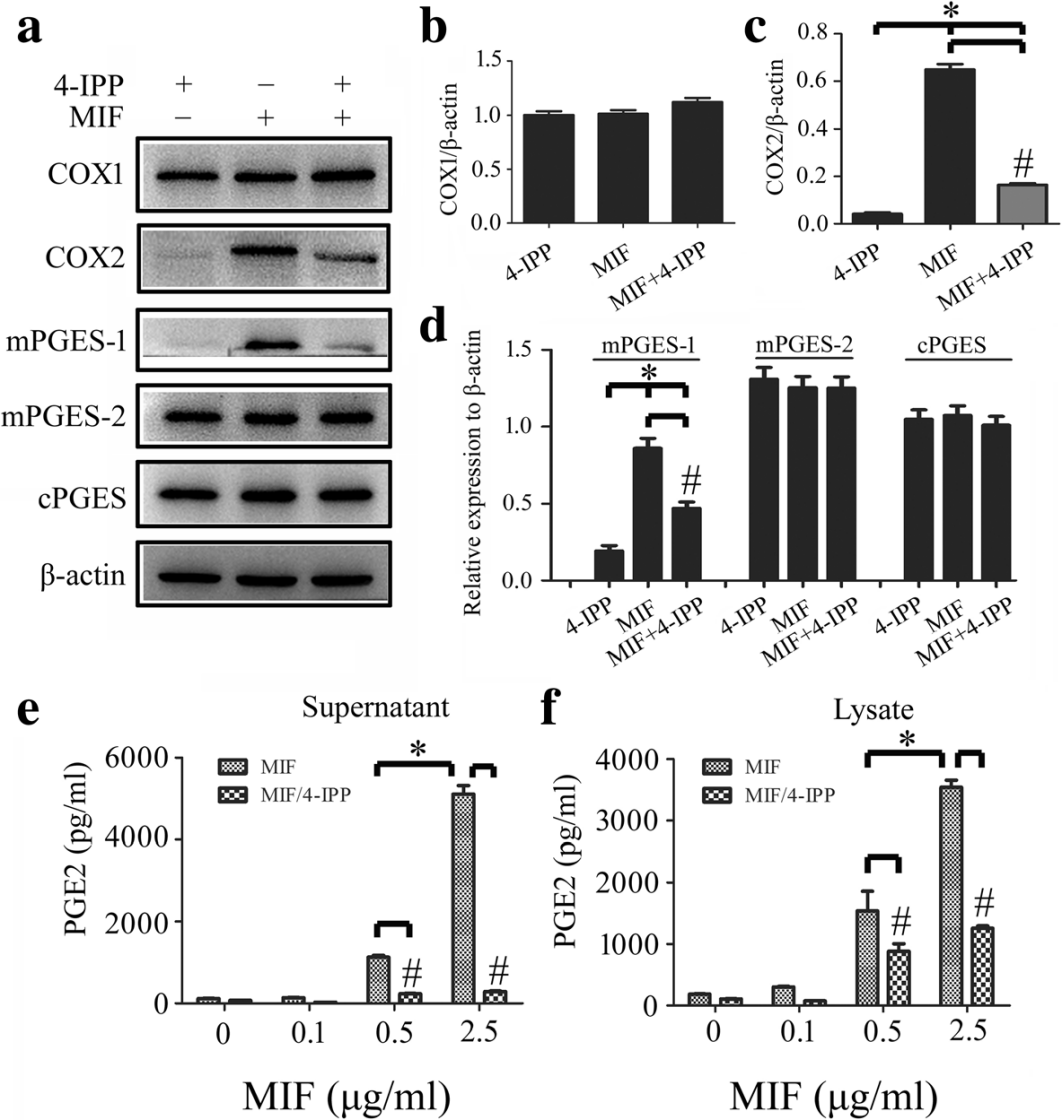
Effects of MIF inhibitor 4-IPP on the expression of PGE_2_ synthesis-related protein. **a** Western blot analysis of COX1, COX2, mPGES-1, mPGES-2, and cPGES following astrocytes treatment with 1 μg/ml recombinant MIF for 24 h in the presence of 100 μM 4-IPP. **b**–**d** Quantification data as shown in (**a**). **e**, **f** Cell supernatants (**e**) and lysates (**f**) were tested by ELISA for the production of PGE_2_, following astrocytes treatment with 0–2.5 μg/ml recombinant MIF for 24 h in the presence of 100 μM 4-IPP. Experiments were performed in triplicates. Error bars represent the standard deviation (*P < 0.05; #*P* < 0.05)

For better insight into the function of MIF on the regulation of COX2/PGE_2_ in astrocytes, primary astrocytes were thus isolated and cultured with purity more than 95% (Fig. 3a). Addition of recombinant MIF protein at concentration of 0–2.5 μg/ml to the astrocytes for 24 h resulted in a dose-dependent elevation of COX2 protein levels, but not of COX1 (Fig. 3b, c). Since the types of prostanoids produced after COX activation are involved in the action of specific prostanoid isomerases [36], mPGES-1, mPGES-2, and cPGES were also determined. Remarkably, mPGES-1, rather than mPGES-2 and cPGES, was induced by MIF treatment (Fig. 3b, d). Addition of 100 μM 4-IPP to the culture in the presence of 1 μg/ml recombinant MIF efficiently blocked the effects of the stimulation (Fig. 4a–d). Correspondingly, the production of PGE_2_ also showed an increase in a dose-dependent manner to MIF stimulation, and addition of 4-IPP was able to attenuate such effects (Fig. 4e, f). An insight of MIF effect on COX2 expression of primary cultured microglia obtained the similar results to those of the astrocytes (Additional file 1: Figure S1). These findings indicate that MIF induces PGE_2_ production in astrocytes in dose-dependent manners relevant to activation of COX2 and mPGES-1.

### Activation of COX2 was essential for MIF-induced production of PGE_2_

**Fig. 5:**
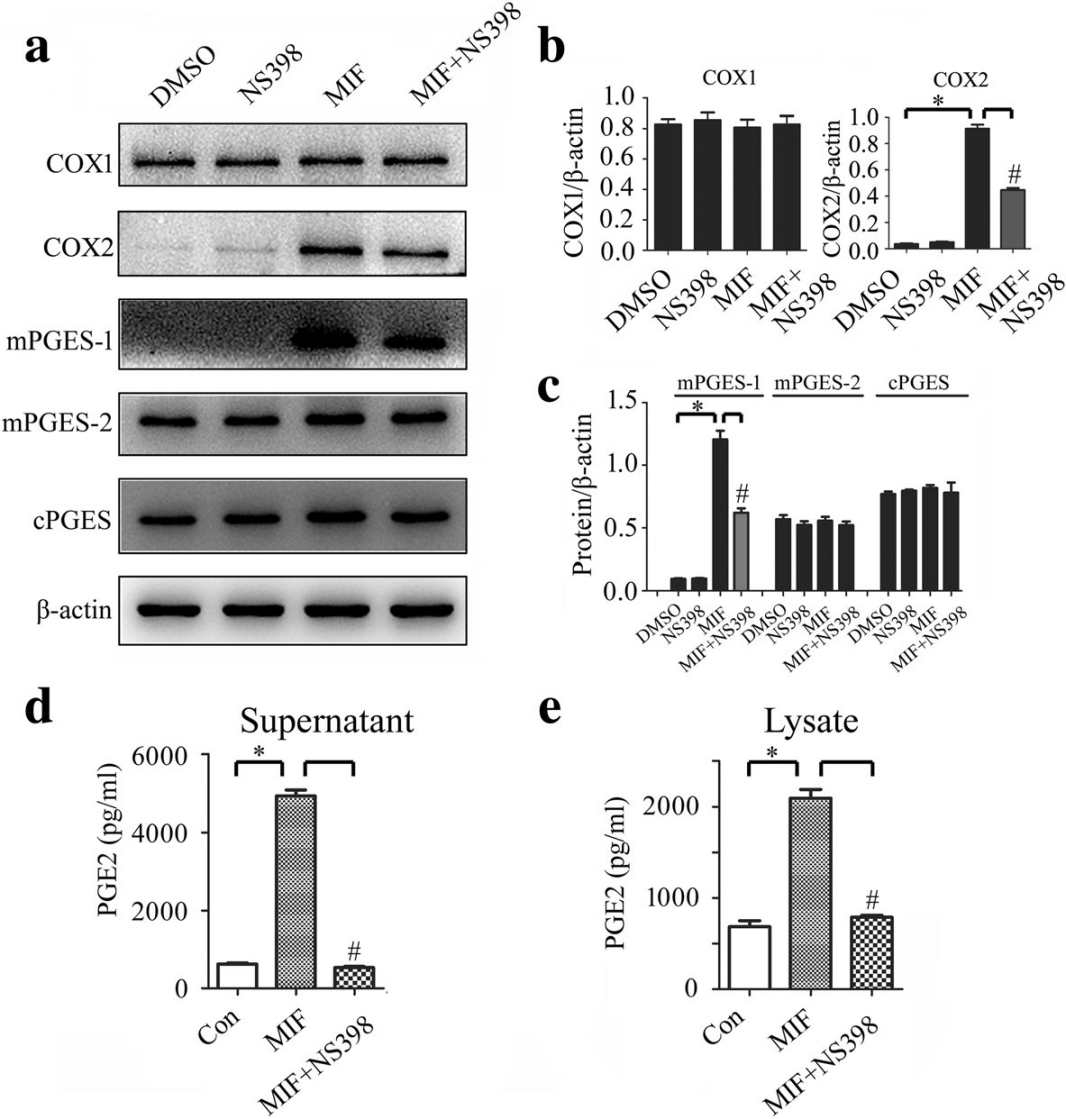
Effects of COX2 inhibitor NS398 on the MIF-induced expression of PGE_2_ synthesis-related protein. **a** Western blot analysis of COX1, COX2, mPGES-1, mPGES-2, and cPGES following astrocytes treatment with 1 μg/ml recombinant MIF for 24 h in the presence of 30 μM NS398. **b**, **c** Quantification data as shown in (**a**). **d**, **e** Cell supernatants (**d**) and lysates (**e**) were tested by ELISA for the production of PGE_2_, following astrocytes treatment with 1 μg/ml recombinant MIF for 24 h in the presence of 30 μM NS398. Experiments were performed in triplicates. Error bars represent the standard deviation (*P < 0.05; #*P* < 0.05)

Now that the expression of COX2 and its downstream mPGES-1 in astrocytes was upregulated in response to stimulation of MIF, the activation of COX2 was possibly essential for the production of PGE_2_. Therefore, the selective COX2 inhibitor NS398 was used to examine the role of MIF in activation of COX2/PGE_2_ pathway. Addition of 30 μM NS398 to the culture in the presence of 1 μg/ml recombinant MIF remarkably decreased the protein levels of COX2 and mPGES-1 in astrocytes, but not of COX1, mPGES-2, and cPGES (Fig. 5a–c). Meanwhile, the production of PGE_2_ in response to MIF stimulation was significantly attenuated by NS398 (Fig. 5d, e). These data indicate that COX2 activation was indispensible for MIF-induced production of PGE_2_.

### MIF activated COX2/PGE_2_ signal pathway through CD74 membrane receptor

Our previous studies have shown that MIF is able to interact with CD74 membrane receptor of astrocytes, which forms a receptor complex with CXCR2 and CXCR4 to elicit intracellular signaling [30]. To clarify whether this interaction can initiate the activation of COX2 and subsequent production of PGE_2_, the siRNA oligonucleotide (siRNA2) which showed nearly 60% knockdown efficiency of CD74 was selected to interfere expression of the receptor [30].

**Fig. 6:**
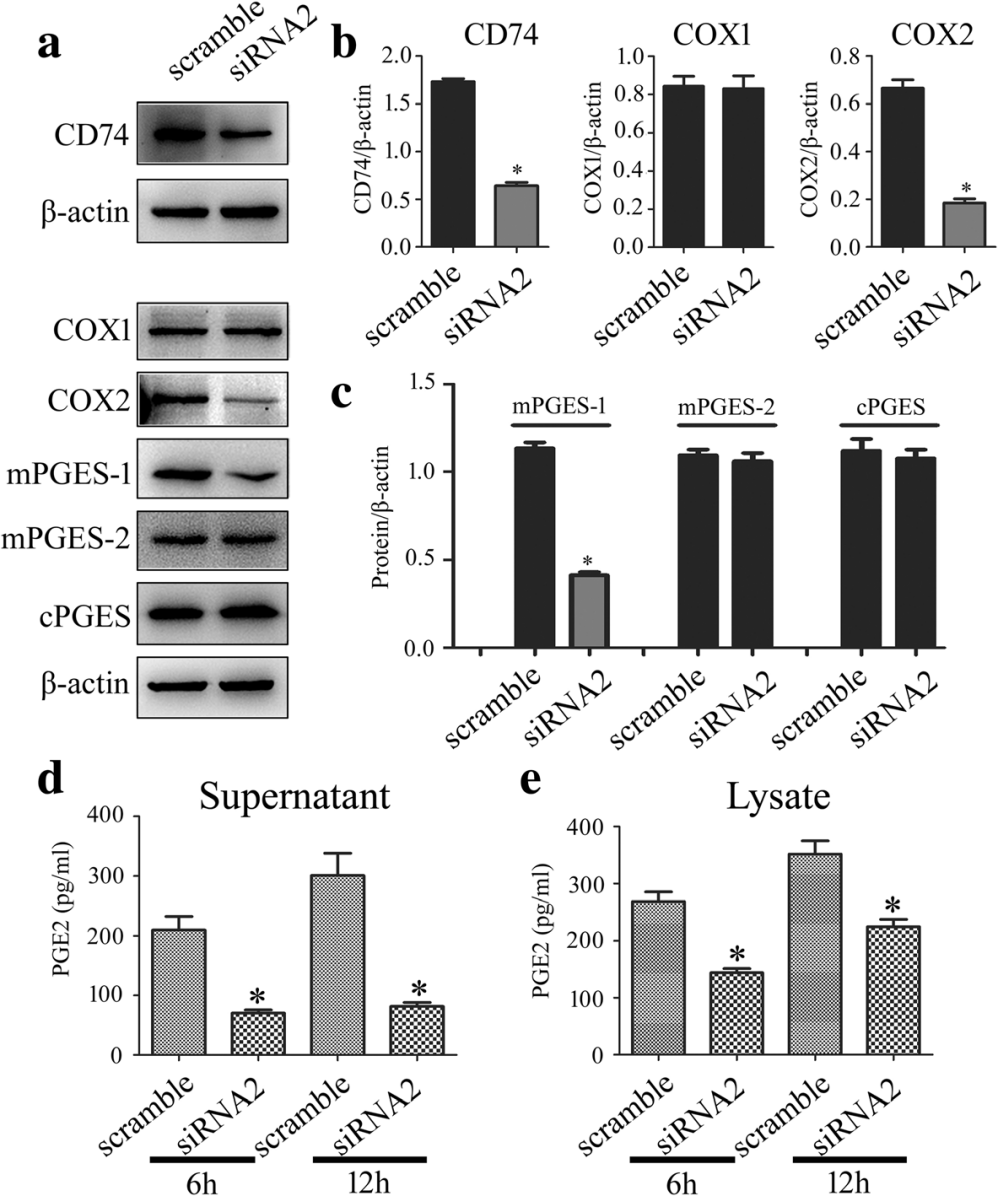
Effects of CD74 knockdown on the MIF-induced expression of PGE_2_ synthesis-related enzymes. **a** Western blot analysis of CD74, COX1, COX2, mPGES-1, mPGES-2, and cPGES following siRNA2 knockdown of CD74 receptor for 48 h, and then treated with 1 μg/ml recombinant MIF at 6 h and 12 h, respectively. A siRNA (scramble) with the same nucleotide composition as siRNA2 but which lacks sequence homology to the CD74 was also designed as negative control. **b**, **c** Quantification data as shown in (**a**). **d**, **e** Cell supernatants (**d**) and lysates (**e**) were tested by ELISA for the production of PGE_2_, following knockdown of CD74 receptor for 48 h, and then treated with 1 μg/ml recombinant MIF at 6 h and 12 h, respectively. Experiments were performed in triplicates. Error bars represent the standard deviation (*P < 0.05)

CD74 receptor of astrocyte was knocked down by siRNA2 for 48 h, followed by the cell treatment with 1 μg/ml recombinant MIF protein for 6 h or 12 h. Results showed that protein levels of COX2 and mPGES-1 remarkably decreased following CD74 interference, while those of COX1, mPGES-2, and cPGES exhibited no changes (Fig. 6a–c). Accordingly, production of PGE_2_ was significantly inhibited by CD74 siRNA following MIF stimulation for 6 h and 12 h, respectively (Fig. 6d, e). These findings indicate that MIF triggers COX2/PGE_2_ signaling through CD74 membrane receptor.

### MIF activated COX2 enzyme through MAPKs pathway

**Fig. 7:**
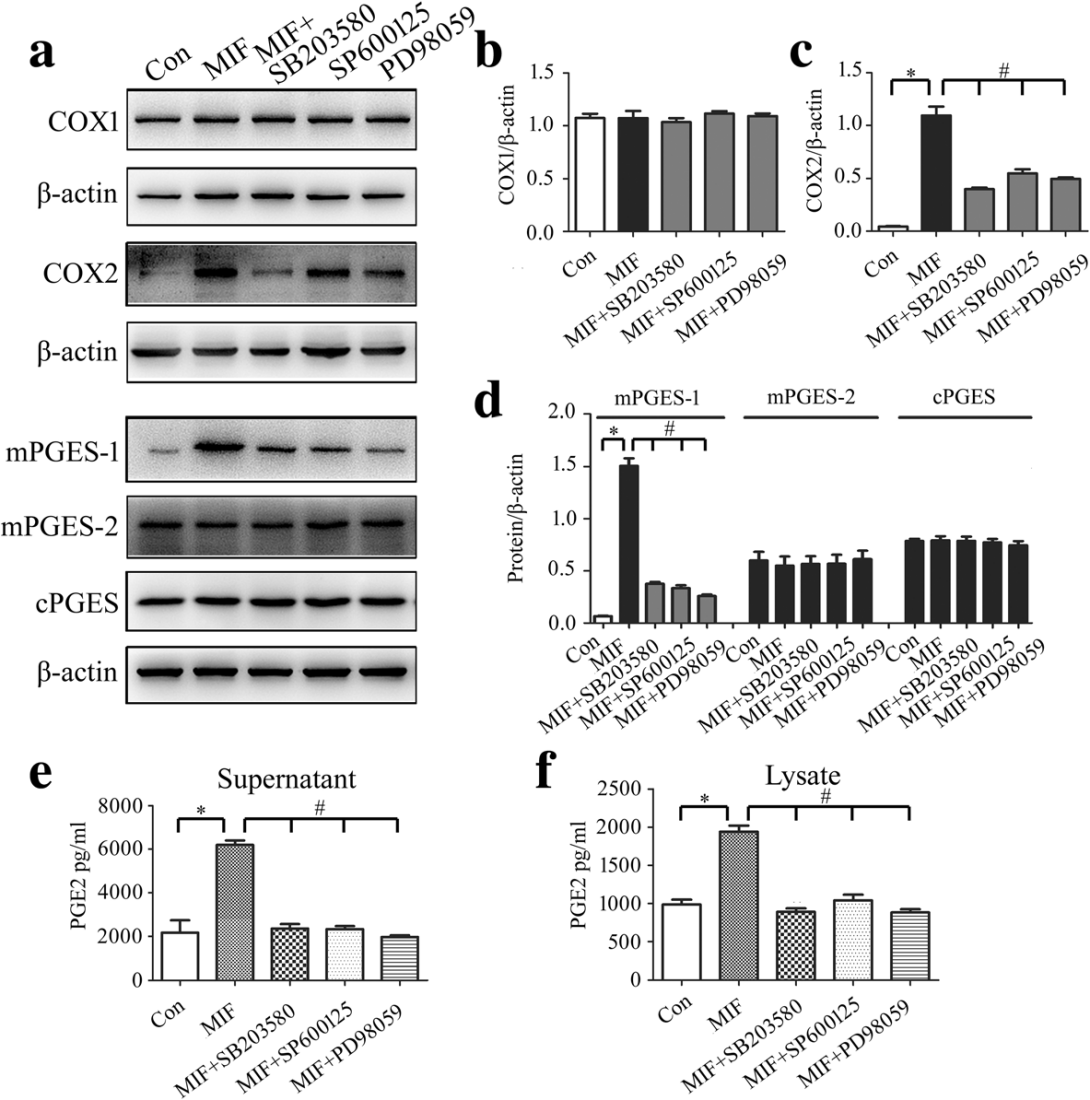
Determination of intracellular signaling associated with regulation of PGE_2_ production. **a** Western blot analysis of COX1, COX2, mPGES-1, mPGES-2, and cPGES following astrocytes treatment with 1 μg/ml recombinant MIF for 24 h in the presence of 10 μM ERK (PD98059), 10 μM P38 (SB203580), or 10 μM JNK (SP600125) inhibitor. **b**–**d** Quantification data as shown in (**a**). **e**, **f** Cell supernatants (**e**) and lysates (**f**) were tested by ELISA for the production of PGE_2_, following astrocytes treatment with 1 μg/ml recombinant MIF for 24 h in the presence of 10 μM ERK (PD98059), 10 μM P38 (SB203580), or 10 μM JNK (SP600125) inhibitor. Experiments were performed in triplicates. Error bars represent the standard deviation (*P < 0.05; #*P* < 0.05)

MIF has been shown to rapidly activate mitogen-activated protein kinases (MAPKs) signaling in fibroblasts, macrophages, and astrocytes [30, 37]. To unveil whether activation of COX2 by MIF was associated with MAPKs, inhibitor of JNK (SP600125), ERK (PD98059), or P38 (SB203580) was used to treat the primary astrocytes in the presence of 1 μg/ml recombinant MIF protein. Determination of PGE_2_ synthesis-related enzymes revealed that induction of COX2 and mPGES-1 by recombinant MIF protein was severely reduced by the inhibitor of JNK, ERK, or P38 (Fig. 7a–d). The production of PGE_2_ was also attenuated by these MAPK inhibitors (Fig. 7e, f). These data indicate that activation of COX2 by MIF is under regulation of MAPKs pathway.

### PGE_2_ released by astrocytes exerted differential impact on the LPS-induced production of inflammatory cytokines

**Fig. 8:**
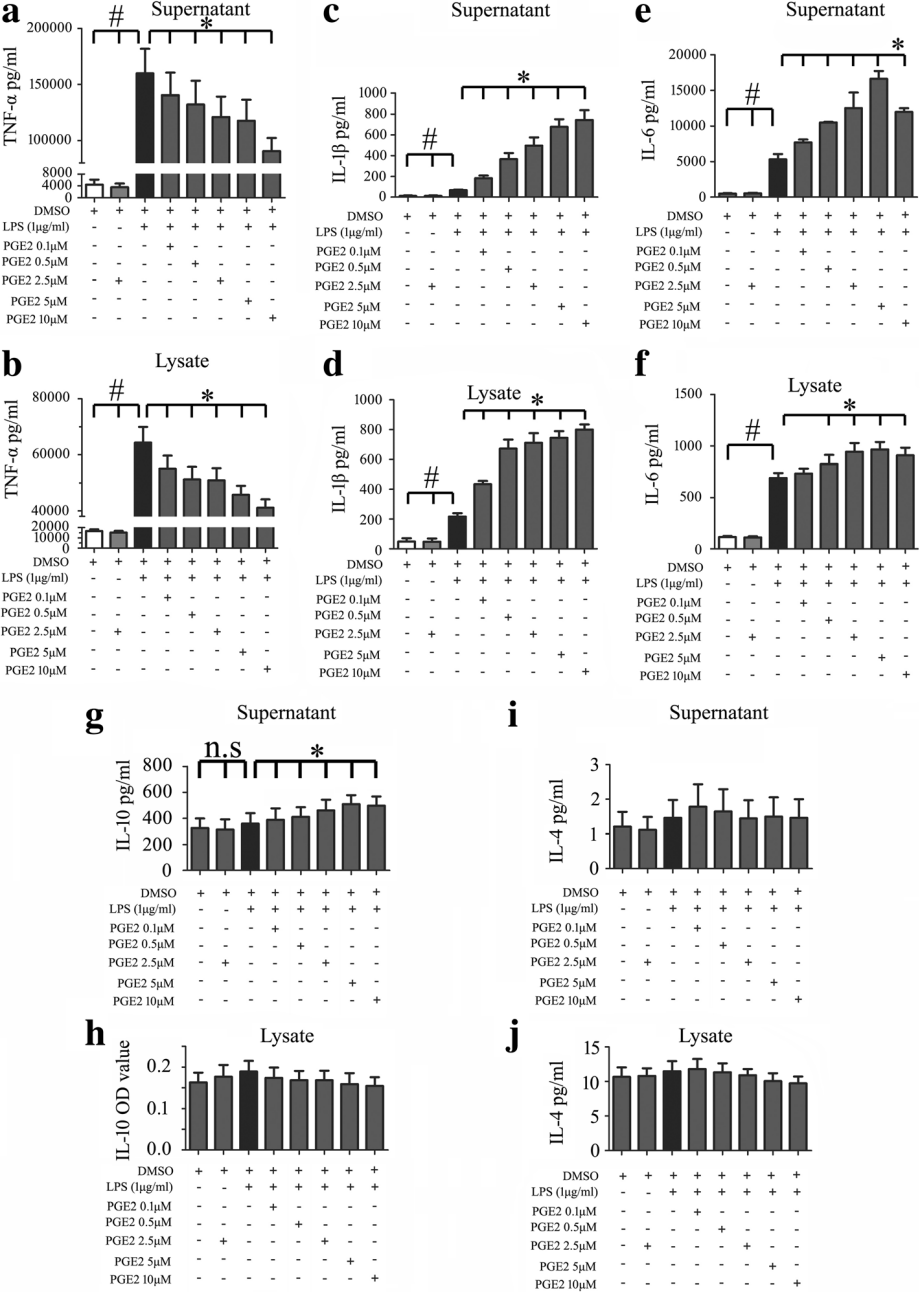
Effects of PGE_2_ on the production of proinflammatory and anti-inflammatory cytokines from LPS-induced macrophages. ELISA assay was used to determine TNF-α (**a**, **b**), IL-1β (**c**, **d**), IL-6 (**e**, **f**), IL-10 (**g**, **h**), or IL-4 (**i**, **j**) production in the supernatants and lysates of macrophage RAW 264.7 following treatment with 1 μg/ml LPS and 0–10 μM PGE_2_, respectively. Experiments were performed in triplicates. Error bars represent the standard deviation (*P < 0.05; #*P* < 0.05)

**Fig. 9:**
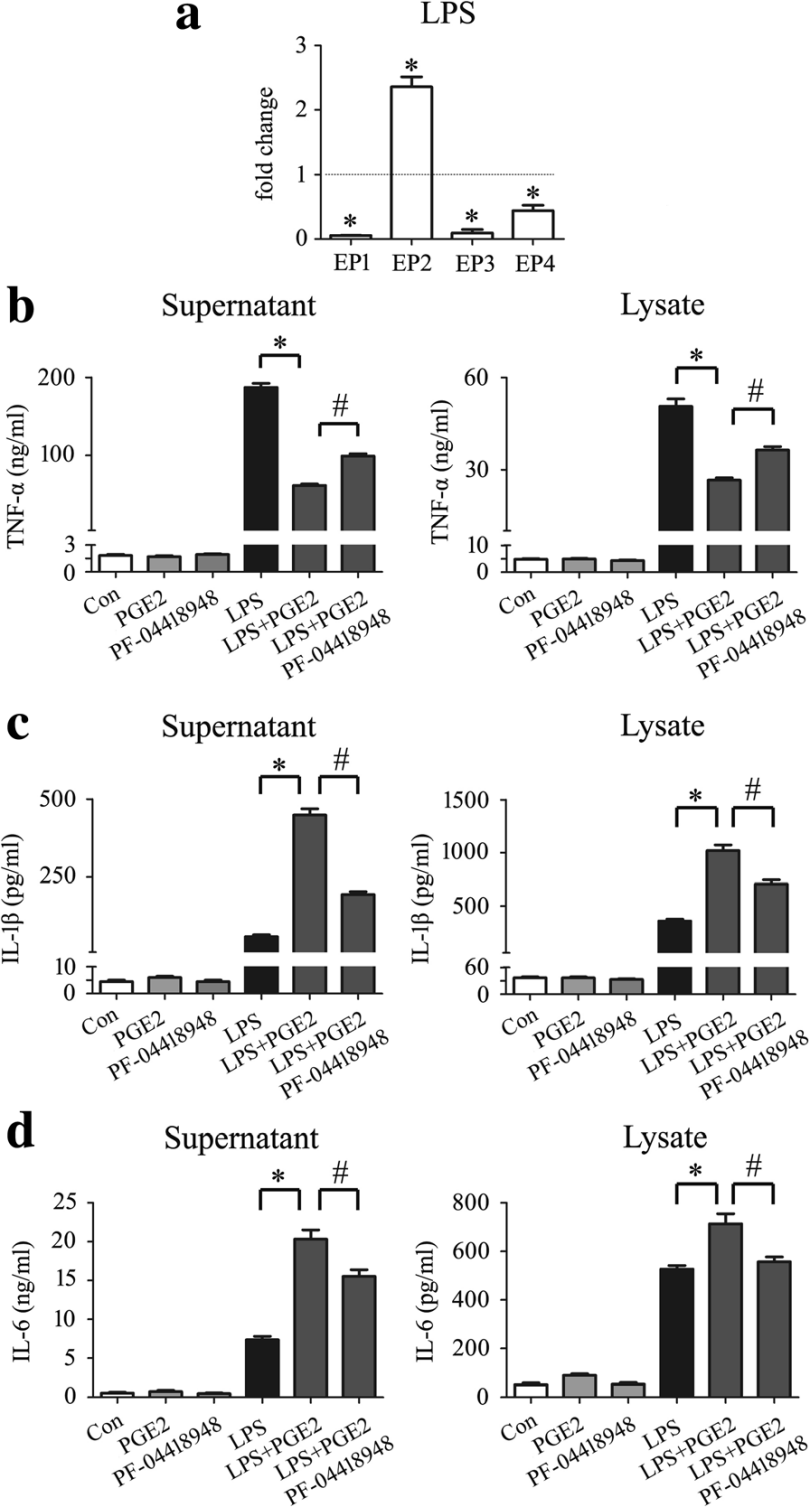
Blocking EP2 receptor attenuated PGE_2_-mediated function on production of inflammatory cytokines. **a** Expression analysis of PGE_2_ receptors by RT-PCR following macrophage treatment with 1 μg/ml LPS for 24 h; ELISA assay was used to determine TNF-α (**b**), IL-1β (**c**), or IL-6 (**d**) production in the supernatants and lysates of macrophage RAW 264.7 following treatment with 1 μg/ml LPS and 2.5 μM PGE_2_ in the presence or absence of 10 μM EP2 inhibitor PF-04418948. Experiments were performed in triplicates. Error bars represent the standard deviation (*P < 0.05; #*P* < 0.05)

Several studies have shown that the production of PGE_2_ during inflammation negatively regulates the production of inflammatory cytokines and chemokines including TNF-α, IFN-β, CCL4, and CCL5 in immune cells [38–41]**.** To understand its influence on the production of injury-related inflammatory cytokines following SCI, macrophage RAW 264.7 cells were challenged with 1 μg/ml Lipopolysaccharide (LPS) for mimicking endogenous DAMPs that induce a broad-spectrum of cytokines through interaction with kinds of PRRs, with or without addition of different concentration of PGE_2_. Assay of cytokines by ELISA showed that PGE_2_ was able to suppress the production of TNF-α, but boost the production of IL-1β and IL6 in the activating macrophages in dose-dependent manner (Fig. 8a–f). Meanwhile, addition of PGE_2_ showed almost no influence on the production of anti-inflammatory cytokine IL-10 and IL-4, as observed from a slight increase of IL-10 protein level in the supernatant (Fig. 8g–j). Because LPS promoted the expression of EP2 receptor remarkably, the EP2-specific antagonist PF-04418948 was thus used to block PGE_2_ pathway (Fig. 9a). Results demonstrated that addition of 10 μM PF-04418948 could attenuate the effects of PGE_2_ on the production of TNF-α, IL-1β, and IL6 (Fig. 9b–d). These findings indicate that PGE_2_ released by astrocytes is able to change the production of proinflammatory cytokines in immune cells.

**Fig. 10:**
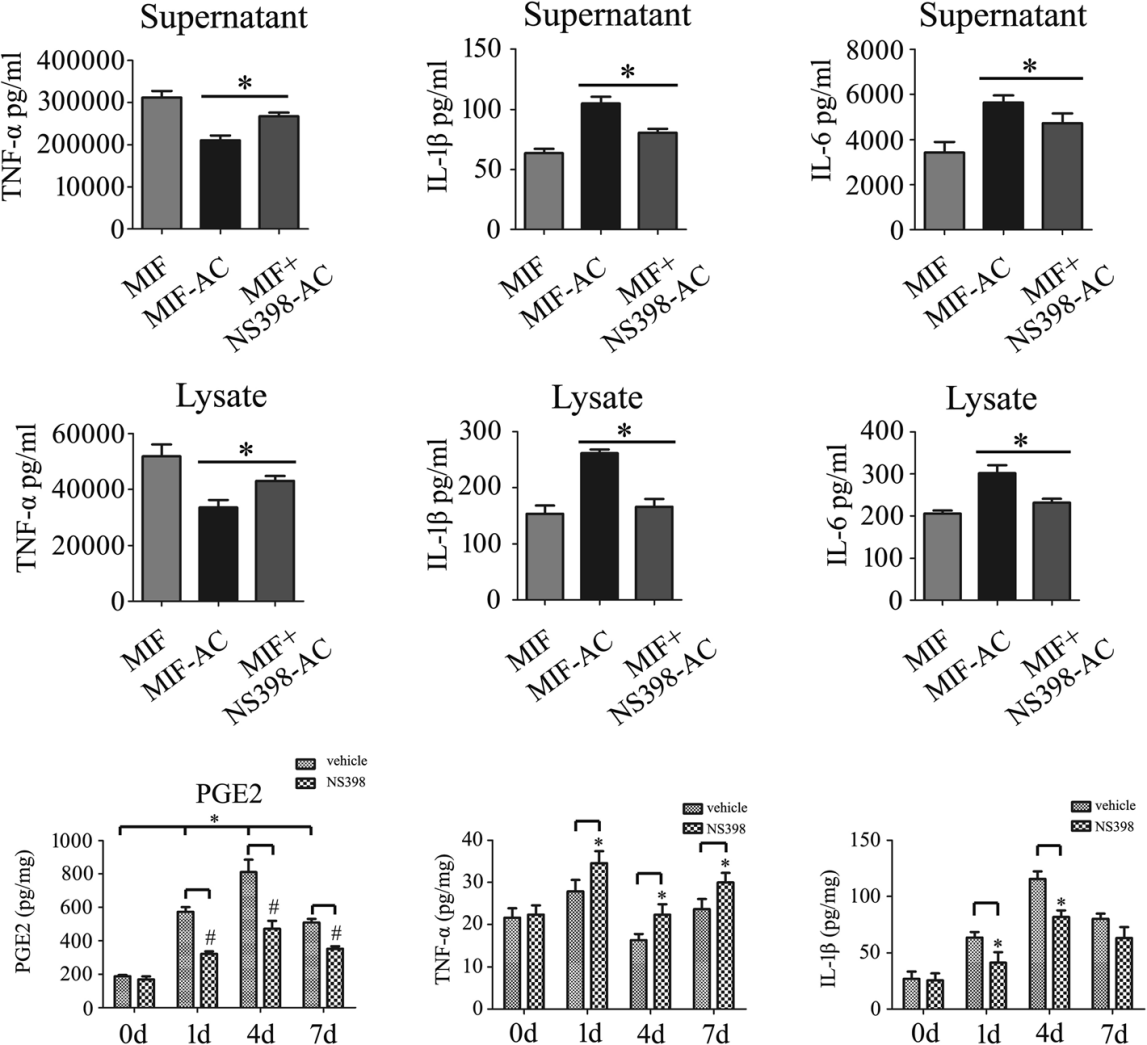
Effects of COX2 inhibitor NS398 on the production of inflammatory cytokines from macrophages in vitro or at spinal cord lesioned site. **a**–**f** ELISA assay determined production of TNF-α (**a**, **b**), IL-1β (**c**, **d**), and IL-6 (**e**, **f**) following macrophages cultured with astrocytes-conditioned medium in the presence of 1 μg/ml LPS for 24 h. The astrocytes were cultured in the presence of 1 μg/ml recombinant MIF with or without 30 μM NS398 for 24 h. **g** Determination of PGE_2_ production at lesion sites of spinal cord following injection of 10 μl 80 mM NS398 at 0 day, 1 day, 4 days, and 7 days, respectively. **h**, **i** ELISA assay determined production of TNF-α (**h**) and IL-1β (**i**) at lesion sites following injection of 10 μl 80 mM NS398 at 0 day, 1 day, 4 days, and 7 days, respectively. Experiments were performed in triplicates. Error bars represent the standard deviation (*P < 0.05; #*P* < 0.05)

To further confirm that astrocyte-released PGE_2_ affects the production of proinflammatory cytokines in macrophages, we cultured astrocytes in the presence of 1 μg/ml recombinant MIF with or without 30 μM NS398 for 24 h. The conditioned medium (CM) from astrocytes-free, COX2 inhibition, or non-inhibition was then collected for macrophage culture in the presence of 1 μg/ml LPS at 24 h. We found that COX2-inhibited CM could increase the production of TNF-α, but significantly decreased the production of IL-1β and IL6 (Fig. 10a–f). These strengthen the conclusion that PGE_2_ influences production of proinflammatory cytokines in macrophages.

To address the pathophysiological function of PGE_2_ following SCI, 10 μl of 80 mM COX2 inhibitor NS398 was injected at lesion sites. Treatment of NS398 significantly decreased PGE_2_ production of injured cord (Fig. 10g). However, the protein level of TNF-α increased, and contrarily, that of IL-1β decreased (Fig. 10h, i). These data indicate that COX2 inhibitor tunes the inflammatory microenvironment through blocking PGE_2_ production.

## Discussion

Following acute CNS injury, CNS elicits a coordinated multicellular inflammatory response that involves glia, neurons, as well as various immune cells [15]. The interaction between these cells constitutes a complex regulatory network of inflammation. For instance, the resident microglia and infiltrating immune cells have been implicated in driving astrocyte-mediated inflammation, while cytokine-stimulated astrocytes also induce migration of immune cells toward lesion sites [42, 43]. It is interesting to note that injured CNS has potential to maximize the preservation of healthy tissue and restricting the spread of cytotoxic inflammation [44]. Astrocytes, the important modulator of milieu, are able to provide benefits to the CNS by phagocytosis of synapses, secretion of neurotrophins, clearance of debris, repair of the BBB, as well as formation of a scar to enclose the necrotic lesion and restrict cytotoxic inflammation [45]. Recently, the metabolites of arachidonic acid lipoxins A4 and B4 have been found to be secreted by astrocytes to promote neuroprotection from acute and chronic injury [46]. In the present study, we have presented that astrocyte-derived PGE_2_ is able to change the components of inflammatory microenvironment, suggesting the dynamic effects of astrocytes on inflammatory homeostasis following SCI.

MIF is constitutively expressed in a variety of immune and non-immune cells of different histogenetic origin. It shows a remarkable functional diversity ranging from activating the production of inflammatory cytokines including TNF-α, IL-1β, IL-6, and IFN-γ to inhibiting p53-mediated cell apoptosis [47]. Given such broad activities, it is not surprising that this pluripotent and pleiotropic cytokine is implicated in acute and chronic inflammatory diseases such as rheumatoid arthritis, asthma, diabetes, sepsis, cardiovascular diseases, and cancer [47–49]. During progression of cancer, MIF has the ability to change tumor microenvironment favorable for tumor aggressiveness. MIF promotes alternative macrophage differentiation that lead to formation of tumor-associated macrophages (TAMs). These TAMs are abundant in the tumor environment and act roles of immunosuppression [50]. In the central nervous system, MIF is inducibly expressed in neurons within the hypothalamus, cortex, and hippocampus to modulate nitric oxide production as well as catecholamine metabolism [51]. Also, it induces neuronal death after compression-induced spinal cord injury [29]. We have shown that MIF is capable of promoting release of chemokine CCL5 from astrocytes, which in turn primarily promote migration of M2-macrophages. In the present study, we displayed that MIF could induce PGE_2_ release from astrocytes to change production of inflammatory cytokines, suggesting multiple mechanisms of MIF in modulation of inflammatory microenvironment following SCI.

PGE_2_ has been found to play a wide range of roles in acute and chronic injury of CNS [52], such as contributing to excitotoxic and ischemic neuronal cell death [53], or neuronal protection [54, 55], depending on its interacting receptor(s). With respect to its function in modulating inflammation in the context of pathophysiological conditions, PGE_2_ has been demonstrated to exert both proinflammatory and immunodepressive actions. During the acute stage of the inflammatory response, PGE_2_ acts as a vasodilator and facilitates tissue infiltration of neutrophils [56], macrophages [57], as well as being a regulator of nociception [58]. However, mounting evidence has also shown that PGE_2_ modulates macrophage activation in part by suppressing the release of cytokines and/or chemokines. For example, PGE_2_ is a negative regulator for LPS-induced production of TNF-α in Epac/PKA-, of IFN-β in Epac/PI3K/Akt-dependent signaling in activated macrophages and during endotoxemia [40, 41]. Within the dying cells, PGE_2_ is induced and released to function as an inhibitory DAMP for inhibiting the expression of genes associated with inflammation, thereby limiting the cell’s immunostimulatory activities [59]. It is noteworthy that PGE_2_ plays an opposing role on IL-1β production from monocytes/macrophages. In primary human monocytes, PGE_2_ boosted LPS-induced IL-1β production [60]. While in human macrophages, PGE_2_ inhibits NLRP3 inflammasome activation through EP4 receptor and intracellular cAMP, leading to reduction in IL-1β secretion [61]. In the present study, we displayed that PGE_2_ attenuated LPS-induced TNF-α, but promoted IL-1β and IL-6 production in macrophage RAW 264.7 cells, suggesting its cell-specific influence on the production of inflammatory cytokines. Although PGE_2_-mediated effects have been found to associate with the resolution of acute inflammation [24], facilitation of tissue regeneration [62], or aggravation of the disease phenotype such as chronic inflammation or cancer [63], its contribution in the injured cord by altering the production of proinflammatory cytokines from macrophages deserves further study. Because reactive astrocytes have potential roles on promoting and restricting inflammation following CNS injury [45], PGE_2_-released by astrocytes might play a role in tuning the microenvironment to maintain homeostasis following SCI.

## Conclusions

Injury-induced MIF activated COX2 expression of astrocytes through binding with CD74 receptor following spinal cord contusion, which in turn promoted the production of PGE_2_. PGE_2_ in turn changes the components of inflammatory microenvironment to maintain homeostasis under neuropathological conditions.

## Additional file

Additional file 1: **Figure S1.** Colocalization of COX2 with IBA-1-positive microglia following spinal cord contusion at 0d and 4d with or without treatment of 4-IPP, as well as examination of 4-IPP effects on COX2 expression. (PDF 187 kb)

## Abbreviations

AA: Arachidonic acid
ANOVA: Analysis of variance
COX: Cyclooxygenases
DAMPs: Damage-associated molecular patterns
ELISA: Enzyme-linked immunosorbent assay
GFAP: Glial fibrillary acid protein
LPS: Lipopolysaccharide
MAPK: Mitogen-activated protein kinases
MIF: Macrophage migration inhibitory factor
mPGES-1: Microsomal PGE synthase-1
MS: Multiple sclerosis
PBS: Phosphate-buffered saline
PGE_2_: Prostaglandin E_2_
SCI: Spinal cord injury
