# Supplementary material for: Macrophage migration inhibitory factor facilitates prostaglandin E2 production of astrocytes to tune inflammatory milieu following spinal cord injury
Source: J Neuroinflammation. 2019 Apr 13;16:85. doi: 10.1186/s12974-019-1468-6 (PMC6461812; doi:10.1186/s12974-019-1468-6)
Supplement: Supplementary file 1 — Figure S1. Colocalization of COX2 with IBA-1-positive microglia following spinal cord contusion at 0d and 4d with or without treatment of 4-IPP, as well as examination of 4-IPP effects on COX2 expression. (PDF 187 kb) [file 12974_2019_1468_MOESM1_ESM.pdf]

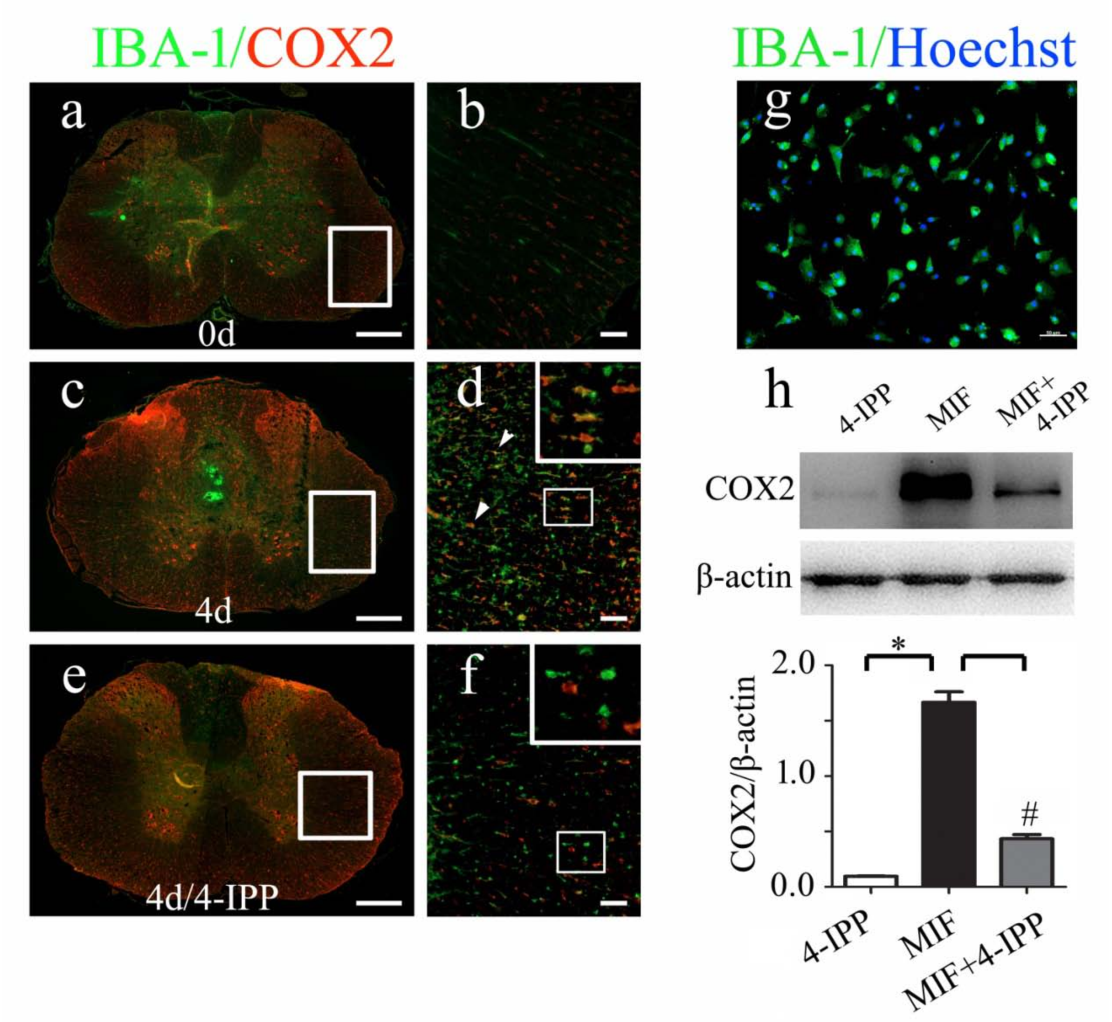

Fig.1 Colocalization of COX2 with IBA-1-positive microglia following spinal cord contusion at 0d and 4d with or without treatment of 4-IPP, as well as examination of 4-IPP effects on COX2 expression. Rectangle indicates region magnified. Arrowheads indicate colocalization of COX2 with astrocytes. Scale bars, 500 μm in (a), (c) and (e); 50 μm in (b), (d), (f) and (g). Error bars represent the standard deviation (*P < 0.05; #P < 0.05).
